# Dissection of stromal and cancer cell-derived signals in melanoma xenografts before and after treatment with DMXAA

**DOI:** 10.1038/bjc.2012.63

**Published:** 2012-03-13

**Authors:** K Henare, L Wang, L-Cs Wang, L Thomsen, S Tijono, C-Jj Chen, S Winkler, P R Dunbar, C Print, L-M Ching

**Affiliations:** 1 Auckland Cancer Society Research Centre, University of Auckland, Private Bag 92019, Auckland, New Zealand; 2 Department of Molecular Medicine and Pathology, University of Auckland, Auckland, New Zealand; 3 Faculty of Medical and Health Sciences, School of Biological Sciences, University of Auckland, Auckland, New Zealand; 4 Maurice Wilkins Centre for Molecular Biodiscovery, University of Auckland, Auckland, New Zealand; 5 New Zealand Bioinformatics Institute, University of Auckland, Auckland, New Zealand

**Keywords:** melanoma, xenografts, stroma, cytokines, DMXAA

## Abstract

**Background:**

The non-malignant cells of the tumour stroma have a critical role in tumour biology. Studies dissecting the interplay between cancer cells and stromal cells are required to further our understanding of tumour progression and methods of intervention. For proof-of-principle of a multi-modal approach to dissect the differential effects of treatment on cancer cells and stromal cells, we analysed the effects of the stromal-targeting agent 5,6-dimethylxanthenone-4-acetic acid on melanoma xenografts.

**Methods:**

Flow cytometry and multi-colour immunofluorescence staining was used to analyse leukocyte numbers in xenografts. Murine-specific and human-specific multiplex cytokine panels were used to quantitate cytokines produced by stromal and melanoma cells, respectively. Human and mouse Affymetrix microarrays were used to separately identify melanoma cell-specific and stromal cell-specific gene expression.

**Results:**

5,6-Dimethylxanthenone-4-acetic acid activated pro-inflammatory signalling pathways and cytokine expression from both stromal and cancer cells, leading to neutrophil accumulation and haemorrhagic necrosis and a delay in tumour re-growth of 26 days in A375 melanoma xenografts.

**Conclusion:**

5,6-Dimethylxanthenone-4-acetic acid and related analogues may potentially have utility in the treatment of melanoma. The experimental platform used allowed distinction between cancer cells and stromal cells and can be applied to investigate other tumour models and anti-cancer agents.

Melanoma accounts for nearly 80% of skin cancer-related deaths, and the incidence of melanoma is increasing rapidly. Melanoma progression involves complex interactions between tumour cells and the non-malignant cells of the tumour stroma (Ch’ng and Tan, 2009). These stromal cells include tumour fibroblasts, which express growth factors such as TGF*β*2 that act on the adjacent tumour cells (van Zijl *et al*, 2009), as well as proteins of the extracellular matrix, which are modified by matrix metalloproteinases derived from tumour cells to regulate tumour invasion and angiogenesis (Kalluri, 2003). Another important component of the melanoma stroma is the vascular endothelial cells, as angiogenesis and a functioning vasculature are necessary in all stages of melanoma progression (Garcia-Barros *et al*, 2003). The interactions of the melanoma cell with its stroma are complex (Ilkovitch and Lopez, 2008) and are incompletely understood. Methodologies that allow the network of messages within the tumour to be dissected would aid in our determination of the mechanisms by which the stroma influences melanoma biology. They will also promote our understanding of how we may disrupt these interactions with novel stromal-targeting agents to impede tumour progression, and how these interactions are altered by traditional chemotherapies that target tumour cells.

Significant benefits for melanoma patients have been obtained through targeted modification of tumour vasculature, immunity and inflammation, such as IFN-*α* (Moschos *et al*, 2006) and isolated limb perfusion of high-dose TNF-*α* therapies (Lienard *et al*, 1992). Ipilimumab, a monoclonal antibody to CTL-4 (Sanderson *et al*, 2005), was recently approved for metastatic melanoma and numerous other immunological approaches for melanoma are also under development (Parmiani *et al*, 2003; Smith *et al*, 2005). In addition, approaches that act to alter the cytokine milieu within tumours, changing the microenvironment from one that is pro-tumour to one that is non-conducive to tumour growth are beginning to make an impact. Examples of these approaches include antagonists of TGF-*β* (Ge *et al*, 2006; Nam *et al*, 2008) and 5,6-dimethylxanthenone-4-acetic acid (DMXAA), a small molecule cytokine inducer (Ching *et al*, 1999a; Joseph *et al*, 1999), developed at the Auckland Cancer Society Research Centre (Rewcastle *et al*, 1991).

In addition to its direct pro-apoptotic action on the tumour vasculature (Ching *et al*, 2002, 2004), DMXAA induces cytokine synthesis *in situ* in the tumour (Ching *et al*, 1999a; Joseph *et al*, 1999). The induced cytokines elicit a cascade of responses: irreversible vascular shutdown, tumour ischaemia and haemorrhagic necrosis (Lash *et al*, 1998) that can result in tumour growth inhibition even in an immunodeficient host. In immunocompetent hosts, the elicited cytokines additionally lead to augmentation of systemic immune cytotoxic T cell activity against the tumour (Jassar *et al*, 2005), an essential component for long-term tumour regressions induced with this class of compounds (Bibby *et al*, 1991; Ching *et al*, 1992).

DMXAA has shown activity against a wide range of preclinical tumour models (Kanwar *et al*, 2001; Jassar *et al*, 2005; Seshadri *et al*, 2006; Seshadri and Ciesielski, 2009), and phase II trials of DMXAA in combination with standard chemotherapy were carried out against ovarian (Gabra and Jameson, 2007), prostate (Pili *et al*, 2010) and lung cancers (McKeage *et al*, 2008). Following promising results in phase II, Novartis sponsored two phase III studies (ATTRACT-1 and ATTRACT-2) of DMXAA in combination with carboplatin and paclitaxel for lung cancer. Although both phase III trials against lung cancer were recently discontinued after interim analysis of the results, DMXAA may potentially have utility against other types of cancers such as melanoma, especially in combination with newer immunological approaches, and provides a paradigm for other derivatives of this class.

In this communication, we describe a multi-modal approach to investigate the distinct effects of a treatment on stromal cells and the cancer cells, using DMXAA and its effects on A375 human melanoma xenografts in nude mice as a proof-of-principle. The experimental platform developed here can be easily applied to other tumour models to better define tumour–stroma crosstalk and to define the mode of action of other stromal-targeting agents.

## Material and Methods

### DMXAA

DMXAA was synthesised as the sodium salt at the Auckland Cancer Society Research Centre (Rewcastle *et al*, 1991) and dissolved fresh for each experiment in saline. 5,6-Dimethylxanthenone-4-acetic acid was administered to mice by intra-peritoneal injection at 25 mg kg^−1^.

### Mice and tumour inoculations

Immunodeficient CD-1 nude mice and RAG-1 knockout (RAG-1^−/−^) mice were bred at the Vernon Jansen Unit, Auckland University. All experiments conformed to local institutional guidelines that meet the standards required by the UKCCCR guidelines. Melanoma lines NZM1, NZM2, NZM3, NZM4 and NZM7, developed from surgical specimens obtained from patients with informed consent, were maintained as previously described (Marshall *et al*, 1993). Xenografts from these lines were initiated by inoculation of 10^7^ cells into the left flank of RAG-1^−/−^ mice. The A375 melanoma line (ATCC no. CRL-1619) was maintained in DMEM media supplemented with 10% FCS, and 10^6^ cells were inoculated subcutaneously into CD-1 nude mice.

### Determination of haemorrhagic necrosis and tumour growth inhibition

Tumours between 8 and 10 mm in diameter were used for haemorrhagic necrosis determinations. Mice with tumours were treated with DMXAA and the tumour excised at 4 or 24 h after treatment. Part of each tumour was fixed in formalin, paraffin-embedded, sectioned and haematoxylin and eosin-stained. Montages of entire tumour sections were acquired (Image Pro PLUS 7.0, Media Cybernetics Inc, Bethesda, MD, USA) at an original magnification × 10 (Nikon TE2000E microscope, Nikon Inc., Kawasaki, Japan). Using Image J 1.45 s software (National Institutes of Health, Bethesda, MD, USA), a grid with 80-*μ*m intersections was overlaid over each montage and the number of grid intersections over necrotic regions as a percentage of the total number of grid intersections was calculated. One entire section from the widest part of the tumour was scored and the mean±s.e.m. of *n*=5 tumours per group was calculated. Data from treated tumours were compared with that of untreated control tumours using one-way ANOVA and Tukey's *post hoc* test and were considered significantly different when *P⩽*0.05.

Growth inhibition studies were initiated when the tumours were 3–4 mm in diameter. Mice (six per group) with tumours were treated with DMXAA and another group was left untreated. Tumours were measured thee times weekly thereafter and tumour volumes were calculated as 0.52*a*^2^*b* where *a* and *b* are the minor and major axes of the tumour. The arithmetic mean±s.e.m. was calculated for each time point and expressed as a fraction of the pre-treatment volume. Delay in tumour re-growth was determined as the number of days required for the treated tumours to return to pre-treatment volumes. Data from treated and untreated tumours were compared using repeated measures two-way ANOVA and Bonferroni *post hoc* test and was considered significantly different when *P⩽*0.05.

### Measurement of cytokines

Tumours between 8 and 10 mm in diameter from untreated or DMXAA-treated mice were excised following cervical dislocation, and weighed and homogenised in 200 *μ*l PBS containing 1 : 100 Sigma Protease Inhibitor Cocktail (Sigma, St Louis, MO, USA). Samples were re-centrifuged and the supernatants stored at −80 °C until assayed for both murine (stromal cell-derived) or human (melanoma cell-derived) cytokines using non-cross-reacting murine and human multiplex cytokine kits (MILLIPLEX MAP Human Cytokine/Chemokine – Premixed 42 Plex, catalogue no. MPXHCYTO-60K-PMX42, and MILLIPLEX MAP Mouse Cytokine/Chemokine – Premixed 32, Plex catalogue no. MPXMCYTO-70K-PMX32, Millipore Corporation, Billerica, MA, USA). Concentration of each cytokine present was read using the Luminex 100 instrument (Luminex Corporation, Austin, TX, USA). The cytokine concentration (picogram per gram of tumour) from three mice per group were expressed as mean±s.e.m. Data between two groups were compared using unpaired Student's *t*-tests or ANOVA, if multiple comparisons were made and were considered significant when the *P*-value was *⩽*0.05.

### Characterisation of tumour-infiltrating leukocytes

Tumours excised at various times after DMXAA treatment were digested with collagenase I (250 U ml^−1^) and dispase (1.66 U ml^−1^; Gibco-URL, Grand Island, NY, USA) at 37 °C for 2 h to yield a single-cell suspension. Erythrocytes were removed using red blood cell lysis buffer (0.15 M ammonium chloride, 10 mM potassium bicarbonate, 0.1 mM EDTA, in sterile MilliQ water (Millipore), pH 7.2–7.4), and the leukocyte fraction was isolated by Ficoll-Paque PLUS (Pharmacia, Uppsala, Sweden) density centrifugation. Leukocytes were labelled with either of two panels of fluorophore-conjugated rat and hamster antibodies described in the Supplementary Methods, or left as unstained controls, in order to identify leukocyte subsets by flow cytometry. Compensation was carried out using a BD CompBeads Anti-Rat and Anti-Hamster Ig *κ*/Negative Control Compensation Particles Set (BD Biosciences, San Jose, CA, USA). Cell populations were analysed using the FACS Aria II cell sorter and Flow Jo 7.6 (TreeStar Inc., Ashland, OR, USA). Labelling was carried out on leukocytes extracted from five tumours pooled for each time point.

### Immunofluorescence staining of tumour sections

Excised tumours were snap frozen and stored at −80 °C until sectioned (7 *μ*m) for immunostaining as previously described (Angel *et al*, 2006, 2009; Lloyd *et al*, 2008). Primary rat anti-mouse antibodies used in these studies were: FITC-labelled anti-CD11b (BD Pharmingen, San Diego, CA, USA), unconjugated anti-F4/80 (Serotec Inc., Raleigh, NC, USA) and unconjugated anti-Ly6G (BD Pharmingen). Sections were first incubated with rat anti-F4/80 or anti-Ly6G, then Alexa Fluor 555-conjugated anti-rat IgG (Molecular Probes, Invitrogen, Camarillo, CA, USA). After blocking with 5% rat serum, sections were probed with FITC-labelled anti-CD11b, subsequently detected with an Alexa Fluor 488-conjugated anti-FITC antibody (Molecular Probes, Invitrogen). Cell nuclei were detected using DAPI stain. Washed sections were mounted with Prolong Gold (Invitrogen) and visualised sequentially using the 350 nm (blue), 470–490 nm (green), and 515–560 nm (red) excitation filters on a Leica DMRE microscope (Leica, Cambridge, UK) and photographed using a Leica DC500 camera (Leica). Sequential images were processed using Portia (CytoCode, Auckland, New Zealand, www.cytocode.com). The amount of autofluorescence was determined using unstained negative controls, and sections stained only with Alexa Fluor 555 anti-rat IgG or Alexa Fluor 488 anti-FITC were used to identify any non-specific binding of the secondary antibodies. These sections were also used to set the input levels for each colour such that the background autofluorescence was reduced to zero, and this setting was applied to every image. Three individual tumours per group were stained and a representative image from each group is presented.

### RNA preparation and microarray hybridisation

RNA was isolated by homogenising frozen tumours in TRIzol reagent (Invitrogen, Carlsbad, CA, USA), resuspended in RNAase-free water (Ambion, Austin, TX, USA), and further purified using the RNA-Midi Kit according to the manufacturer's instructions (Qiagen Inc., Valencia, CA, USA). The final product was eluted in RNAse-free water, and RNA integrity assessed by capillary gel electrophoresis using an Agilent Technologies 2100 Bioanalyser (Agilent technologies UK Limited, Cheshire, UK), and frozen at −80 °C until analysis. The RNAs were used as templates to prepare Biotin-labelled complex cRNAs that were hybridised separately to both human U133plus2 and mouse 430 v2.0 Affymetrix gene chips, according to standard Affymetrix protocols (Affymetrix, High Wycombe, UK).

### Analysis of microarrays

*(a) Analysis of the cross-hybridisation potential of Affymetrix probe sets*: Since xenografts contain RNAs derived from two different species, a control experiment was carried out to estimate the potential for cross-species hybridisation of specific human and mouse probe sets in our experimental context. Control RNAs from both human (cultured A375 cells) and mouse (a mixture of tissues from lymph node, skin and vascular regions of subcutaneous fat) were hybridised to both human U133plus2 and mouse 430 v2 gene chips. From these human-on-human, mouse-on-human, mouse-on-mouse and human-on-mouse microarray data, we generated lists of probes sets that had the potential to hybridise across species, as described in the Supplementary Methods. This approach identified 7176 mouse 430 v2 probe sets and 7797 human U133plus2 probe sets that may potentially cross-species hybridise, representing 16% and 14% of the probe sets on the human and mouse gene chips, respectively (Supplementary File 1). Conversely, we identified a second set of transcripts that were unlikely to cross-species hybridise. These probe sets: (i) were not in the lists above of probe sets with potential for cross-species hybridisation and, (ii) in addition, the RNA from the other species was shown to be present and therefore to be capable of producing a cross-species hybridising signal. This approach identified 6105 mouse 430 v2 probe sets and 7163 human U133plus2 probe sets that were unlikely to cross-species hybridise (Supplementary File 1).

*(b) Analysis of xenograft transcripts*: Mouse and human data from untreated and DMXAA-treated tumours (*n*=5 each) were normalised at the probe level using the RMA algorithm from the Bioconductor *Affy* package. The mouse and human gene chips were normalised separately and data were log_base2_-transformed. The RMA normalised data were not significantly altered by exclusion of the probes/probe sets estimated to be likely to cross-species hybridise, therefore, all probe sets were used for further analysis. The Ingenuity Pathways Analysis (IPA) database (http://www.ingenuity.com) was used to identify putative activation of molecular pathways by DMXAA. To assess the likelihood of identifying gene lists that appeared to be enriched for molecular pathways by chance alone, we performed permutation analyses as described in the Supplementary Methods. Our gene array data are available from the Gene Expression Omnibus web repository, accession number GSE26308. All manipulation of the data was performed within the bioinformatic environment R (www.r-project.org), using modules from the Bioconductor Affy and LIMMA packages.

### Species-specific quantitative RT–PCR (qRT–PCR)

Complementary DNA was produced from oligodT-primed mRNA using the Omniscript RT Kit (Qiagen). Primers for the putative DMXAA-regulated mRNAs *CCL3*, *CCL4* and *CCL7* and ‘housekeeping controls’ *YWHAZ* and *PPIA* were designed with a 3′ base difference between species and their species-specificity confirmed using RT–PCR of control RNAs from mouse and human cells. Melting curves were calculated to ensure purity of PCR products. Cycle thresholds for *CCL3/MIP-1α*, *CCL4/MIP-1β* and *CCL7/MCP-3* were normalised for total RNA content using the average of the *YWHAZ* and *PPIA* signals. This analysis was performed in two separate experiments of two and three xenografts, respectively.

## Results

### Antitumour activity of DMXAA on A375 melanoma xenograft histology and growth

**Figure 1:**
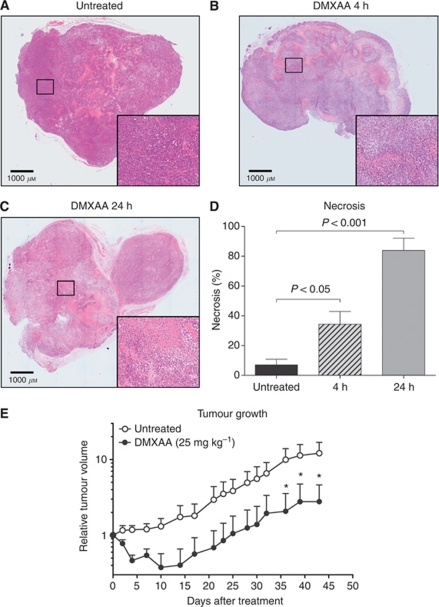
Antitumour effects of DMXAA against A375 melanoma xenografts in CD-1 nude mice. Degree of haemorrhagic necrosis in a representative montage of haematoxylin and eosin-stained sections from A375 xenografts untreated (**A**), 4 h (**B**) and 24 h after DMXAA (25 mg kg^−1^) (**C**). Insets show the field of view in the rectangle at original magnification × 10. Percent haemorrhagic necrosis determined in montaged sections (**D**). Bars represent mean±s.e.m. (*n*=5 tumours per group). *P*-values determined by one-way ANOVA and Tukey's *post hoc* test showing significant differences between treated and untreated groups. Growth of subcutaneous A375 xenografts untreated (open circles) and after single dose of DMXAA (25 mg kg^−1^; closed circles) (**E**). Mean±s.e.m. of six mice per group. Growth curves compared using repeated measures two-way ANOVA and Bonferroni correction for multiple comparisons. Asterisks denote significant difference between treated and untreated groups; ^*^P⩽0.05.

A multi-modal approach to analysing the effects of DMXAA on human A375 melanoma xenografts in nude mice was carried out. First, we examined the effects of DMXAA on the histology of the A375 melanoma xenografts. A single dose of DMXAA at 25 mg kg^−1^ induced 34±9% at 4 h (Figures 1B and D), and 84±8% at 24 h (Figures 1C and D) haemorrhagic necrosis that was significantly greater than that observed in untreated (7±4%) xenografts (Figures 1A and D). In a separate experiment, we compared the growth of A375 xenografts untreated, and following treatment with single dose of DMXAA at 25 mg kg^−1^. 5,6-Dimethylxanthenone-4-acetic acid treatment delayed A375 re-growth by 26 days, with significant differences in tumour volume obtained 36 days post treatment compared with untreated controls (Figure 1E).

### Effects on stromal cell infiltrates following DMXAA treatment

**Figure 2:**
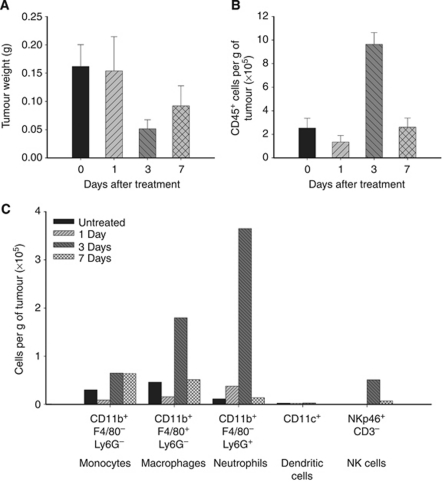
DMXAA induced changes in leukocyte populations in A375 xenografts. Tumour weight (mean±s.e.m. from *n*=6 per group) (**A**) and CD45^+^ leukocytes (mean±s.e.m. from two independent experiments, each using six tumours per time point) (**B**) in untreated and DMXAA-treated (25 mg kg^−1^) A375 xenografts. Composition of CD45^+^ leukocytes extracted from A375 xenografts, untreated and 1, 3, and 7 days after DMXAA treatment (**C**). Each bar represents data collected from five tumours pooled for each time point from a single experiment.

The leukocyte content of the A375 melanoma xenografts was next examined. CD45^+^ leukocytes per gram tumour weight increased nearly five-fold 3 days after treatment with DMXAA (Figure 2B), when tumour weight had decreased by 70% (Figure 2A). Multi-channel FACS analysis was used to characterise the leukocyte population in xenografts (Figure 2C). Neutrophil (CD11b^+^Ly6G^+^ cells) numbers showed the greatest change and by 3 days had increased over 10-fold (Figure 2C). Monocytes and macrophages decreased in numbers over the first 24 h, but after 3 days, cells with the phenotype of mature macrophages (CD11b^+^F4/80^+^) had increased five-fold. A sharp peak of NKp46^+^ NK cells was seen only on day 3. CD11c^+^ dendritic cells were not detected before or after DMXAA treatment (Figure 2C). (See Supplementary Figure 1 for gating and dot plots.)

**Figure 3:**
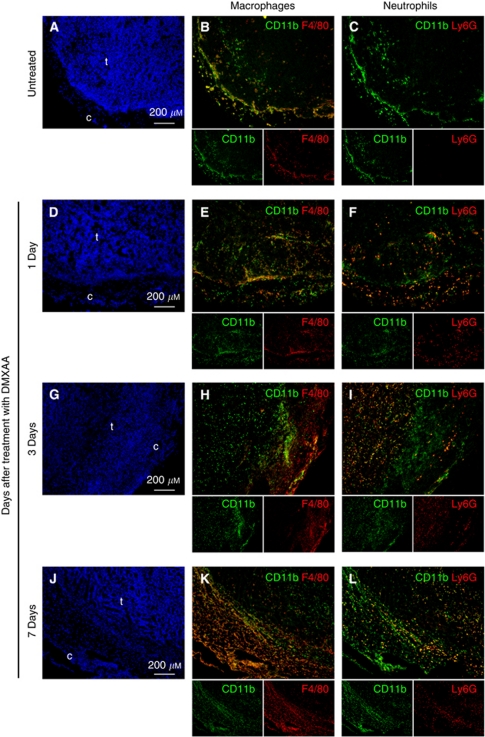
Spatial distribution of macrophages and neutrophils in A375 xenografts following DMXAA treatment. Sequential sections of A375 xenografts untreated (**A**, **B**, **C**), 1 day (**D**, **E**, **F**), 3 days (**G**, **H**, **I**) or 7 days (**J**, **K**, **L**) after DMXAA treatment at 25 mg kg^−1^. DAPI-stained (**A**, **D**, **G**, **J**) to locate nuclei of cells in capsule (c) or tumour parenchyma (t); immunofluorescently stained for CD11b^+^ (green), F4/80^+^ (red) and double positives (yellow/orange) indicative of mature macrophages (**B**, **E**, **H**, **K**); or immunofluorescently stained for CD11b^+^ (green), Ly6G^+^ (red) and double positives (yellow/orange) indicative of neutrophils (**C**, **F**, **I**, **L**). All images were acquired at an original magnification × 10. The large panels (**B**, **C**, **E**, **F**, **H**, **I**, **K**, **L**) represent the merged image, with the individual green and red images shown in the smaller panels underneath. Representative section from *n*=3 tumours per group.

The spatial distribution of the neutrophils and macrophage/monocyte populations in untreated and treated A375 xenografts was examined using double immunofluorescence labelling of sequential cryosections (Figure 3). Untreated A375 xenograft sections showed the presence of CD11b^+^F4/80^+^ macrophages in the capsule and periphery of the tumours (Figure 3B), and no Ly6G^+^ neutrophils (Figure 3C). One day after DMXAA treatment, both macrophages (CD11b^+^F4/80^+^; Figure 3E) and neutrophils (CD11b^+^Ly6G^+^; Figure 3F) were seen within the tumour parenchyma. On day 3, the majority of the CD11b^+^ cells in the tumour also expressed the Ly6G marker indicating that the infiltrate was primarily neutrophils (Figure 3I), and these cells were still present 7 days after treatment (Figure 3L). The immunofluorescence studies carried out using three tumours per time point (Figure 3) confirm those obtained with flow cytometry analyses of the leucocyte populations carried out using five pooled tumours for each time point (Figure 2C).

### Dissection of the cytokine response induced in stromal cell and melanoma cells

**Figure 4:**
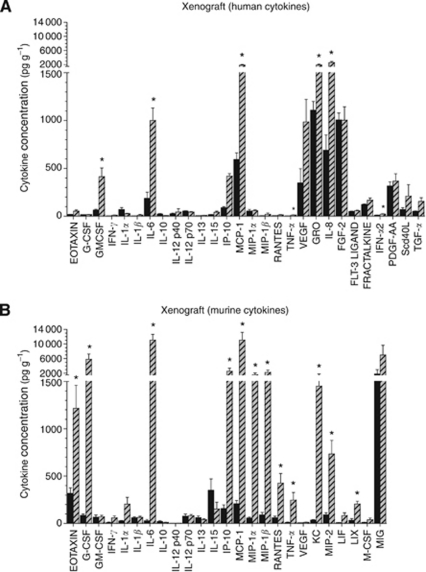
Cytokines upregulated by DMXAA in A375 xenografts. Human melanoma cell-derived cytokines (**A**) or murine, stromal cell-derived cytokines (**B**) in A375 xenografts untreated (black bars) or 4 h after DMXAA (25 mg kg^−1^) treatment (hatched bars). Mean±s.e.m. of *n*=3 mice. Asterisks denote statistically significant difference compared with untreated controls (^*^P⩽0.05 by unpaired *t*-tests).

Cytokines in A375 xenografts before and 4 h after treatment were assayed using human and murine multiplex cytokine panels. A number of human cytokines were detected above lower limits of detection of the assay in the untreated A375 xenografts: most notably, MCP-1, GRO, IL-8 and FGF-2 (Figure 4A). Following DMXAA treatment, significant increases in both human (Figure 4A) and murine cytokines (Figure 4B) were observed. Human, melanoma cell-derived cytokines elevated by DMXAA included GM-CSF, IL-6, IP-10, MCP-1, GRO and IL-8 (Figure 4A). Murine, stromal cell-derived cytokines elevated by DMXAA included eotaxin, G-CSF, IL-6, IP-10, MCP-1, MIP-1*α*, MIP-1*β*, RANTES, and TNF-*α*, KC, MIP-2, and LIX (Figure 4B). 5,6-Dimethylxanthenone-4-acetic acid treatment induced higher concentrations and a greater number of stromal cell-derived cytokines compared with A375-derived cytokines.

**Figure 5:**
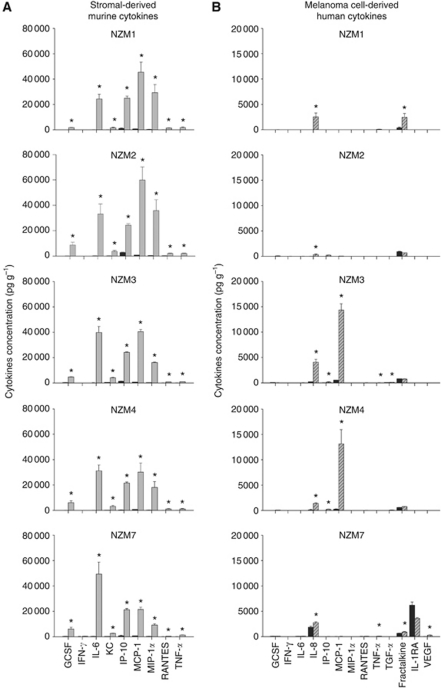
Murine (**A**) and human (**B**) cytokines in melanoma xenografts in RAG-1^−/−^ mice, untreated (black bars) or 4 h after DMXAA (grey bars (**A**); hatched bars (**B**)). Mean (pg g^−1^)±s.e.m. (*n*=3 per group). Asterisks denote statistically significant difference compared with untreated controls (*P*⩽0.05 by unpaired *t*-tests).

When the cytokine profiles produced in xenografts from five other human melanoma lines, NZM1, NZM2, NZM3, NZM4 and NZM7 derived from New Zealand melanoma patients (Marshall *et al*, 1993) were compared, DMXAA was shown to induce a consistent panel of stromal cell-derived cytokines: G-CSF, IL-6, IP-10, KC, MCP-1, MIP-1*α*, RANTES and TNF-*α* in all five NZ melanoma xenografts (Figure 5A). In contrast, the melanoma cell-derived cytokines produced constitutively or in response to DMXAA varied between cell lines (Figure 5B). Again, higher concentrations and a larger panel of stromal-derived murine cytokines were observed compared with melanoma-derived human cytokines.

### Global mRNA expression in tumour and stromal cells

**Figure 6:**
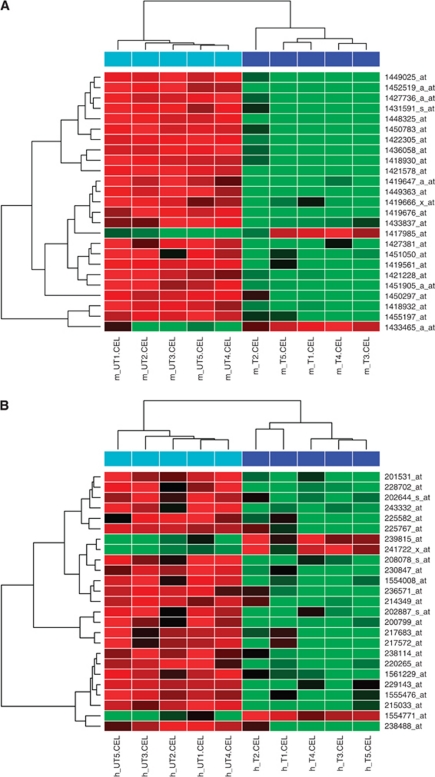
Heatmaps illustrate the differential abundance of most significantly differentially expressed 25 probe sets (rows) when *P*⩽0.05 and fold change of ⩾1.5 up or down are selected in mouse arrays (**A**) and human arrays (**B**). The red and green colours represent a continuous expression range across the four gene chips (red=low and green=high). To make visualisation easier, the gene chips and the probe sets that had similar features were grouped together in columns and rows, respectively, based on hierarchical clustering.

We next examined the separate transcriptomes of the tumour cells (human) and stromal cells (murine) by microarrays in the five treated and five untreated A375 xenografts. We identified the global mRNA profiles of the stromal and A375 melanoma cells of the untreated xenografts and how these profiles were affected by DMXAA. Labelled RNAs from each of five untreated and five DMXAA-treated xenografts were hybridised to both human (U133plus2) and mouse (430v2) Affymetrix microarrays. The problem of potential cross-species hybridisation (Harrell *et al*, 2008) was addressed in a control microarray experiment (see Materials and Methods), where we generated a list of probe sets that clearly hybridised to RNA from the other species, and a list of probe sets that were unlikely to cross-species hybridise (Supplementary File 1). Linear models (LIMMA) were used to identify and rank DMXAA-induced or -repressed RNAs. Human and murine probe sets with a differential expression *P*-value <0.05 and a fold change ⩾1.5 up or down were selected for further analysis (listed in Supplementary Files 2 and 3). To illustrate these changes, the most significantly differentially expressed 25 probe sets in human and mouse are shown in heat maps in Figure 6.

### RNAs and molecular pathways activated by DMXAA in A375 xenografts

**Figure 7:**
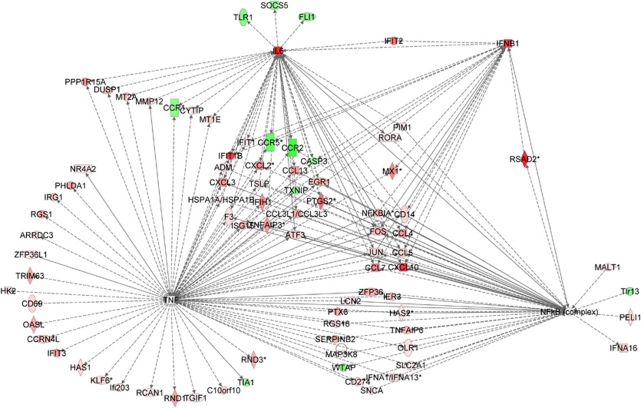
The RNAs regulated in stromal cells (mouse) by DMXAA constitute members of NF-*κ*B/TNF-*α*/IL-6/IFN-*β* molecular pathways more than would be expected by chance. Graphs are from the IPA database. Red indicates that a molecule was upregulated by DMXAA, green indicates downregulated by DMXAA, and no colour indicates that a molecule was not significantly regulated by DMXAA.

In stromal cells, according to the criteria described above, DMXAA appeared to downregulate 98 probe sets, of which 49 could be mapped to known RNA transcripts, and 49 represented uncharacterised ESTs. 5,6-Dimethylxanthenone-4-acetic acid appeared to upregulate 140 probe sets, of which 18 were duplicate probe sets representing the same transcripts, and 49 represented uncharacterised ESTs. Analysis using the Gene Ontology database through the Gather web tool suggested that several transcripts regulated by DMXAA in stromal cells encoded molecules associated with defence/immune responses. Twenty-nine of the murine DMXAA-regulated RNAs were associated with the GO category GO:0006952 for defence response. This is significantly more transcripts of this type than expected due to chance (Bayes Factor=11 and permutation *P*=0.02). Stromal cytokine transcripts induced by DMXAA included *cxcl10 (IP-10), ccl4 (MIP-1β), il6* and *ccl2 (MCP-1)*, which concurred with the protein data for these transcripts measured using Luminex multiplex cytokine assays (Table 1). Analysis using the IPA database suggested that the DMXAA-regulated stromal cell RNAs were enriched for the targets of: NF-*κ*B transcription factors, TNF-*α* pathways, IFN-*β* pathways and IL-6 pathways (Figure 7). A permutation analysis (see Supplementary Methods) indicated that the intersections observed between our list of DMXAA-regulated stromal genes, and the targets of NF-*κ*B/TNF-*α*/IL-6/IFN-*β* pathways, were unlikely to have occurred by chance, with empirical *P*-values of 0.02 and 0.05, 0.03 and 0.04, respectively. Although not induced themselves by DMXAA at the RNA level, NF-*κ*B, TNF-α, IL-6 and IFN-*β* have several downstream targets that are differentially expressed between control and DMXAA-treated tumours. This coordinated change in abundance of RNAs downstream of each of the NF-*κ*B/TNF-*α*/IL-6/IFN-*β* pathways is consistent with DMXAA regulating the activity of these pathways in stromal cells, either directly or indirectly.

**Figure 8:**
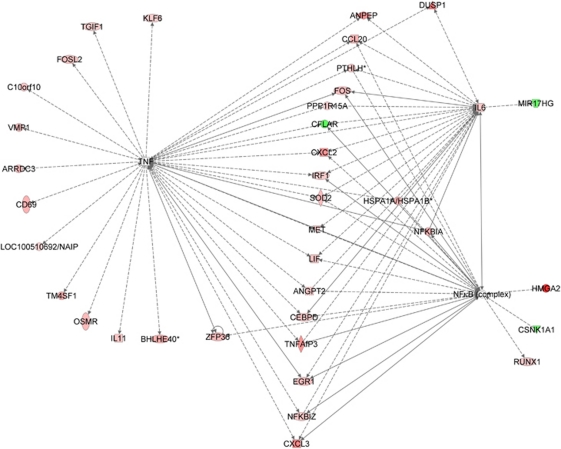
The RNAs regulated in tumour cells (human) by DMXAA constitute members of NF-*κ*B/TNF-*α*/IL-6 molecular pathways more than would be expected by chance. The features in the graph have the same meaning as those in Figure 7.

DMXAA also appeared to regulate mRNA abundance in the human A375 melanoma cells within the xenografts. 5,6-Dimethylxanthenone-4-acetic acid downregulated 30 probe sets, of which 12 could be mapped to known RNA transcripts and 18 represented uncharacterised ESTs. 5,6-Dimethylxanthenone-4-acetic acid appeared to upregulate 117 probe sets, of which 4 were duplicate probe sets representing the same transcripts and 57 represented uncharacterised ESTs. As with DMXAA-regulated stromal RNAs, several cancer cell-derived RNAs, including *CCL2 (MCP-1), IL-8, IL-6, CXCL1 (Melanoma Growth Stimulating Activity/GRO1)*, also concurred with the protein data (Table 2). As in stroma, DMXAA appeared to induce in A375 cells the expression of several RNAs downstream of NF-*κ*B, TNF-*α* and IL-6 pathways (Figure 8). Permutation analyses suggested that the intersections observed between our list of DMXAA-regulated A375 melanoma cell genes and the targets of these three pathways were unlikely to have occurred by chance (*P*=0.04, 0.03 and 0.02, respectively).

As some of the molecules described above were flagged as probe sets that may exhibit cross-species hybridisation, as proof of our principle of the species-specific (and therefore A375 melanoma-specific and stroma-specific) microarray analysis, we tested whether the stromal cell-specific induction by DMXAA of three chemokines, *ccl3 (MIP-1α), ccl4 (MIP-1β)* and *ccl7 (MCP-3*), could be confirmed using species-specific qRT–PCR. Both human- and mouse-specific oligonucleotide primers for *CCL3*, *CCL4* and *CCL7* and ‘housekeeping controls’ for normalisation (*YWHAZ* and *PPIA*, *CYC1, RPLPO* and *ACTB)* were designed with a 3′ base corresponding to a base that differs between species. All were confirmed by qRT–PCR to amplify only from template RNAs from a single species. Quantitative RT–PCR using these primers suggested that all three chemokines were induced by DMXAA in the stromal cells but not significantly in the A375 melanoma cells (Supplementary Figure 3).

## Discussion

Despite the growing clinical use of drugs that target the tumour stroma, we have little understanding of the precise mechanisms of action of many of these agents. We also have relatively little understanding of the molecular pathways passing between the malignant cells and the stromal cells that these agents perturb. Therefore, we urgently require experimental systems to assess how a drug affects the cancer cell and stromal cell compartments, and how these effects lead to changes in the entire tumour tissue. To address this challenge we have performed an integrated, multi-modal study using A375 melanoma xenografts, which has provided an understanding of how the stromal-targeting agent DMXAA, distinctly affects the melanoma and the stromal cells on multiple levels. Remarkable consistency was observed – for example, between active pro-inflammatory molecular pathways, RNAs encoding cytokines and the cytokine proteins themselves, and between these cytokines and the leukocyte subsets that infiltrated into the tumours. The microarray data provided new information that receptors for almost all of the DMXAA-induced cytokines are expressed by stromal cells irrespective of DMXAA treatment, whereas the RNA encoding CCRL2, a receptor for CCL19 (MIP-3*β*), was additionally upregulated by DMXAA. We also saw clear examples of the complexity of tumour biology, where TNF-*α*/IL-6 pathways were activated by DMXAA in both A375 cells and stromal cells. This appeared to cause the transcription factors associated with these pathways such as NF-*κ*B to promote the expression of their transcriptional targets, including numerous molecules that are themselves involved in TNF-*α*/IL-6 signalling. Technical considerations of our pathway analysis approach using cross-species hybridisation are described in the Supplementary Discussion.

The consistency between RNA, protein and immunohistological data allows us to build up a multi-level model of the effects of DMXAA on tumour tissue, in this first report of growth inhibition induced with DMXAA against human melanoma xenografts. A single dose of DMXAA induced widespread haemorrhagic necrosis (Figures 1C and D), and a 26-day delay in tumour re-growth (Figure 1E). The neutrophil accumulation seen in Colon 38 tumours in immunocompetent mice after DMXAA treatment (Wang *et al*, 2009), was also seen here in A375 xenografts in immunodeficient nude mice (Figure 3). Treatment of murine sarcomas with combretastatin-4-phosphate (CA4P), another vascular disrupting agent, is also associated with neutrophil accumulation (Parkins *et al*, 2000), which could in part represent an inflammatory response to tumour necrosis induced by anti-vascular agents. However, this simple inflammatory response is unlikely to fully account for the massive neutrophil influx observed with DMXAA-treated tumours. We have shown that DMXAA can directly induce leukocytes in culture to produce cytokines with neutrophil chemotactic activities (Wang *et al*, 2006), and the neutrophil infiltration in tumours seen following DMXAA treatment could therefore be initiated by the induced chemoattractants (e.g., MIP-1*α* and IP-10). The chemokines induced following DMXAA treatment are strikingly similar to those induced following TGF-*β* blockade that result in polarisation of tumour-associated neutrophils from pro-tumour to antitumour phenotypes (Fridlender *et al*, 2009). Therefore, it will be interesting to examine the phenotype of neutrophils isolated from DMXAA-treated tumours.

When xenografts of five primary human melanoma cell lines were treated with DMXAA, the cytokines produced by the stromal cells (Figure 5A) were, in all cases, similar to those induced in stromal cells in A375 xenografts (Figure 4B), and similar to that in Colon 38 tumours in syngeneic immunocompetent mice (Wang *et al*, 2009). This suggests that the effect of DMXAA on murine stroma is consistent, irrespective of the tumour cell type or the mouse strain. In contrast, the cytokines induced by DMXAA in the various melanoma lines as xenografts were variable (Figure 5B), and a melanoma-specific cytokine signature is not evident. Whether or not the various NZMel xenografts exhibit differential sensitivities to DMXAA treatment, and whether their responsiveness can be linked to their profile of cytokine production has yet to be investigated.

In A375 xenografts, we observed an overlapping but distinct effect of DMXAA on cytokine protein production by melanoma cells and the stromal cells, which was also seen at the level of gene expression in the microarrays (Tables 1 and 2) and in three cases verified with RT–PCR (Supplementary Figure 3). Several stromal-derived cytokines were, however, shown to be significantly upregulated at the protein level but not at the RNA level (Table 1). For example, although mRNA for murine TNF-*α* and G-CSF were not significantly induced, these cytokines were highly induced at the protein level 4 h after DMXAA treatment (Table 1). These results provide strong indication of post-transcriptional regulation by DMXAA in the production of these cytokines. Stromal-derived G-CSF is the most notable of the cytokines that appears to be upregulated by DMXAA at the post-transcriptional level (Table 1). A 68-fold increase in murine G-CSF protein was obtained in A375 xenografts after DMXAA treatment with no change in mRNA expression. Recent studies show that G-CSF promotes metastasis and tumour vascularisation through a number of different mechanisms that include mobilisation of endothelial precursor cells (Voloshin *et al*, 2011), and mobilisation of granulocytes (Kowanetz *et al*, 2010). Elevated levels of plasma G-CSF, VEGF and SDF-1 are detected in cancer patients treated with CA4P (Shaked *et al*, 2009). The production of these cytokines in mice with tumours following treatment with CA4P and its second generation derivatives has been suggested to contribute to the rapid re-vascularisation of the treated tumours, thereby diminishing the antitumour effects of this class of vascular disrupting agents (Shaked *et al*, 2009; Voloshin *et al*, 2011). In *G-CSF-R*^*−/−*^ mice, SDF-1 was downregulated and mobilisation of endothelial precursor cells was not observed compared with wild-type control mice following treatment with a CA4P derivative, indicating that SDF-1 is secondarily involved in G-CSF-induced mobilisation of endothelial precursor cells (Shaked *et al*, 2009). In another study, inhibition of SDF-1 signalling decreased influx of TIE2-expressing, pro-tumour macrophages into murine mammary tumours and increased the efficacy of CA4P treatment (Welford *et al*, 2011). In this study, protein levels of SDF-1was not measured, VEGF was unchanged, but G-CSF was markedly increased in A375 xenografts following DMXAA treatment (Figure 4B). The A375 melanoma is non-metastatic and non-invasive, and it would be of interest to compare the rates of tumour re-vascularisation and re-growth following treatment with DMXAA and CA4P in an invasive, metastatic tumour model in relation to their differential induction of the pro-angiogenic cytokines.

The analysis of the xenografts at the whole transcriptome level using microarrays allows inference of the activity of molecular pathways (Creighton *et al*, 2003, 2005; Hull *et al*, 2008). We identified pro-inflammatory gene sets including NF-*κ*B targets induced by DMXAA in both tumour and stromal cells (Figures 6 and 7). For example, 27% of 125 mRNAs upregulated in stroma following DMXAA treatment had NF-*κ*B response elements in their promoters. This strengthens our previous suggestions that DMXAA activates the NF-*κ*B pathway (Ching *et al*, 1999b; Woon *et al*, 2003; Wang *et al*, 2006). Consistent with previous reports that IFN-*β* has an important role in the antitumour effects of DMXAA (Roberts *et al*, 2007, 2008), mRNAs encoding *IFNA1*, *IFNB1* as well as known IFN targets (Figures 6 and 7) were shown here to be induced. Our studies also indicated that DMXAA activated TNF-*α*/IL-6 pathways in both cancer and stromal cells and caused transcription factors downstream of these pathways, such as NF-*κ*B, to promote the expression of their transcriptional targets, including molecules that are themselves involved in TNF-*α*/IL-6 signalling. When we estimated the potential for tumour–stroma crosstalk based on expression above background of RNAs encoding stroma-derived factors and their tumour cell receptors, we found numerous potential stroma-tumour interactions. We also noted the induction by DMXAA of potential interactions involving stroma-derived IFNB1 and IL-6, consistent with the analysis of differential expression and the histological data. We also noted that A375 cells in xenografts downregulate metabolic enzymes including oxidative stress response genes and upregulate components of the extracellular matrix, consistent with the relatively hyperoxic state and lack of three dimensional matrix structure of melanoma cells grown in tissue culture. This supports the investigation of drug effects using a xenograft model rather than simple tissue culture studies.

The studies were designed to provide insights into the molecular pathways and target(s) for DMXAA, which are still not completely defined. Multiple targets are likely to be involved in order to account for the numerous and distinct effects that DMXAA has on different cell types. Recent efforts from our laboratory using the approach of photoaffinity labelling (Palmer *et al*, 2007; Brauer *et al*, 2010) identified more than 30 cellular proteins that were found to interact with the azido-analogue of DMXAA. Essentially, all the labelled proteins were oxidisable proteins, implicating a role for redox signalling in the action of DMXAA (Brauer *et al*, 2010). We suggest that under physiological conditions, enzymes catalyse the one-electron oxidation of DMXAA to form the benzyl radical and subsequent production of reactive oxygen species (Ching *et al*, 2010). Identification of the cellular enzymes that oxidise DMXAA to initiate redox signalling is a high priority in our laboratory, as these enzymes may also be regarded as biochemical target(s) of DMXAA.

In summary, this study has provided proof-of-principle of an integrated multi-modal approach to dissect molecular signals, pathways and tissue structure in cancer and stromal cells. In doing so, we have shown that DMXAA can target melanoma xenografts by inducing complex cytokine signalling cascades that are distinct but overlapping between cancer cells and the various stromal cell populations and lead to haemorrhagic necrosis in the tumour tissue. The use of a xenograft model allowed the differential effects of DMXAA on stroma (murine origin) to be distinguished from its effects on the cancer cells (human origin). Although this approach cannot be directly applied to the use of the drug on a clinical setting, we believe the integrated approach described here will be useful for the study of other experimental tumour models and drugs to provide insights into potential crosstalk between soluble proteins secreted by stromal cells and their receptors on cancer cells and *vice versa* (see Supplementary results) that will aid in our understanding of the action of a drug in the clinical setting.

## Figures and Tables

**Table 1:** Comparison between protein and RNA data for DMXAA-regulated molecules in stroma (mouse)

**Protein name**	**Gene symbol**	**Protein fold change**	**RNA fold change**
IP-10	*cxcl10*	15.8	13.4
IL-6	*il6*	372.2	7.7
MIP-1*β*	*ccl4*	24.7	4.7
MIP-1*α*	*ccl3*	26.9	4.1
MCP-1	*ccl2*	52.8	3.1
MIP-2	*cxcl2*	8	2.6
KC	*cxcl1*	41.4	2.2
RANTES	*ccl5*	7	2.1
MIG	*cxcl9*	3.9	2
IL-1*β*	*il1b*	1	1.5
TNF-*α*	*tnf*	23.1	1.4
IFN-*γ*	*ifng*	6.4	1.3
LIX	*lix*	6.1	1.3
EOTAXIN	*ccl11*	3.8	1.2
IL-4	*il4*	1.9	1.2
IL-15	*il15*	0.4	1.1
IL-1*α*	*il1a*	7.6	1.1
M-CSF	*csf1*	3.6	1.1
IL-3	*il3*	1.3	1.1
IL-10	*il10*	0.5	1.1
IL-2	*il2*	1.1	1.1
LIF	*lif*	9	1.1
IL-17	*il17a*	1.3	1
G-CSF	*csf3*	67.9	1
IL-13	*il13*	0.7	0.9
IL-5	*il5*	5.3	0.9
IL-12	*il12a*	1.1	0.9
IL-9	*il9*	0.5	0.8

**Table 2:** Comparison between protein and RNA data for DMXAA-regulated molecules in tumour cells (human)

**Protein name**	**Gene symbol**	**Protein fold change**	**RNA fold change**
MCP-1	*CCL2*	3.1	2.7
IL-8	*IL8*	3.7	2.4
GRO	*CXCL1*	1.7	2.4
VEGF	*VEGF*	2.8	1.9
GMCSF	*CSF2*	6.5	1.8
IL-6	*IL6*	5.4	1.5
MIP-1*β*	*CCL4*	4.2	1.3
TNF-*α*	*TNFA*	3.1	1.3
IL-1*α*	*IL1α*	0.4	1.2
FGF-2	*FGF2*	1	1.2
FLT-3 L	*FLT3LG*	1.1	1.2
IP-10	*CXCL10*	4.8	1.2
TGF-*α*	*TGFA*	3	1.2
RANTES	*CCL5*	1.9	1.1
MCP-3	*CCL7*	1.5	1.1
MIP-1*α*	*CCL3*	1.1	1.1
IFN-*α*2	*IFNα2*	2	1.1
IL-12P40	*IL12P40*	1.7	1.1
IL-1*β*	*IL1β*	3.2	1.1
IL-15	*IL15*	2.5	1.1
IL-2RA	*IL2RA*	1.8	1.1
FRACTALKINE	*CX3CL1*	1.4	1.1
PGGF	*PDGF*	1	1.1
IL-17	*IL17*	1	1.1
IL-3	*IL3*	1	1.1
G-CSF	*CSF3*	1.1	1
IFN-*γ*	*IFNγ*	2.6	1
EGF	*EGF*	2.8	1
IL-4	*IL4*	1	1
IL-1RA	*IL1RA*	2.2	1
IL-13	*IL13*	1.7	1
TNF-*β*	*TNFB*	1	1

## Supplementary Material

Supplementary Table 5

Supplementary Infomation
