# Supplementary figures and images for: Dissection of stromal and cancer cell-derived signals in melanoma xenografts before and after treatment with DMXAA
Source: Br J Cancer. 2012 Mar 13;106(6):1134–47. doi: 10.1038/bjc.2012.63 (PMC3304430; doi:10.1038/bjc.2012.63)

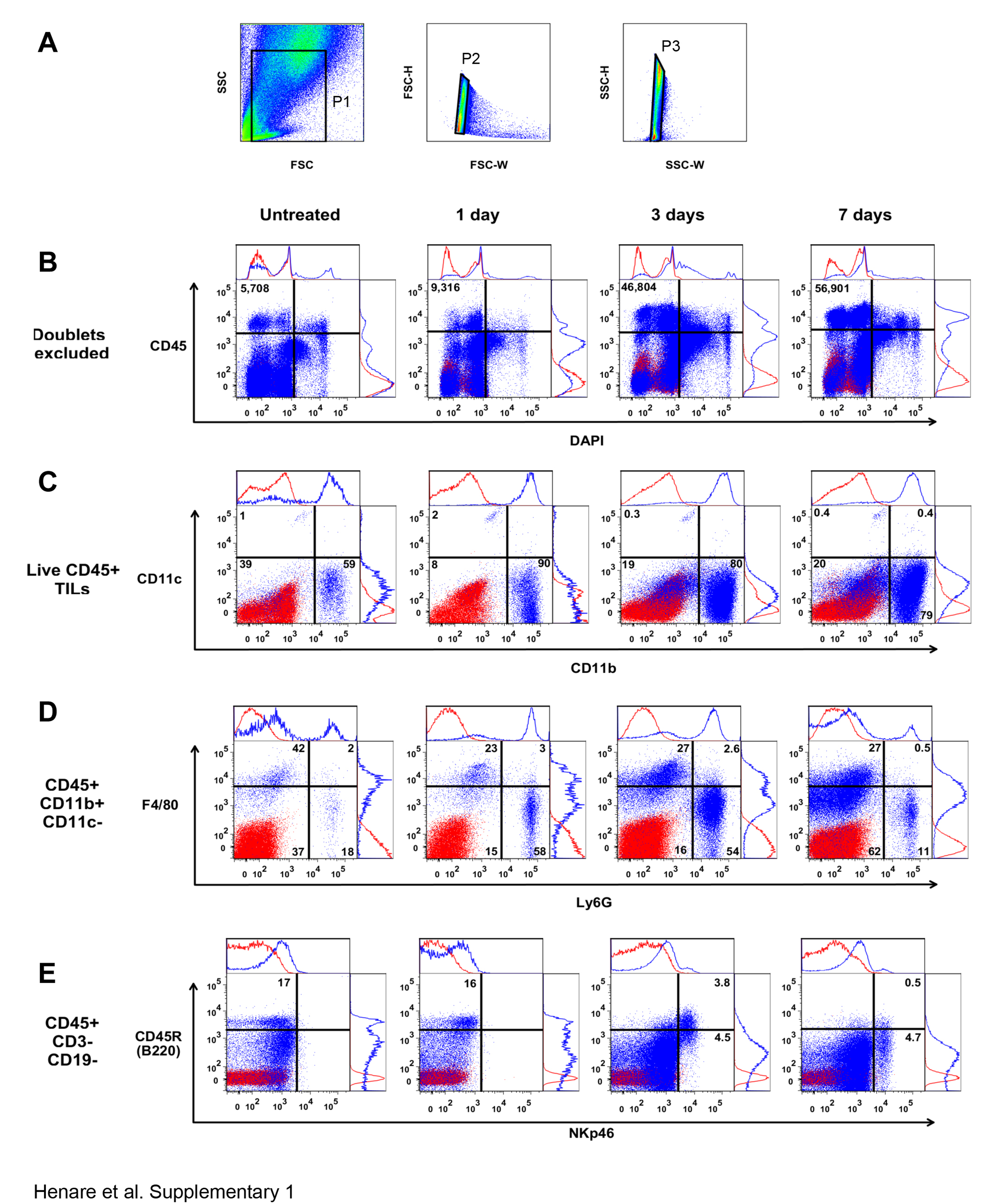

Supplement: Supplementary Figure 1 [file bjc201263x1.tif]

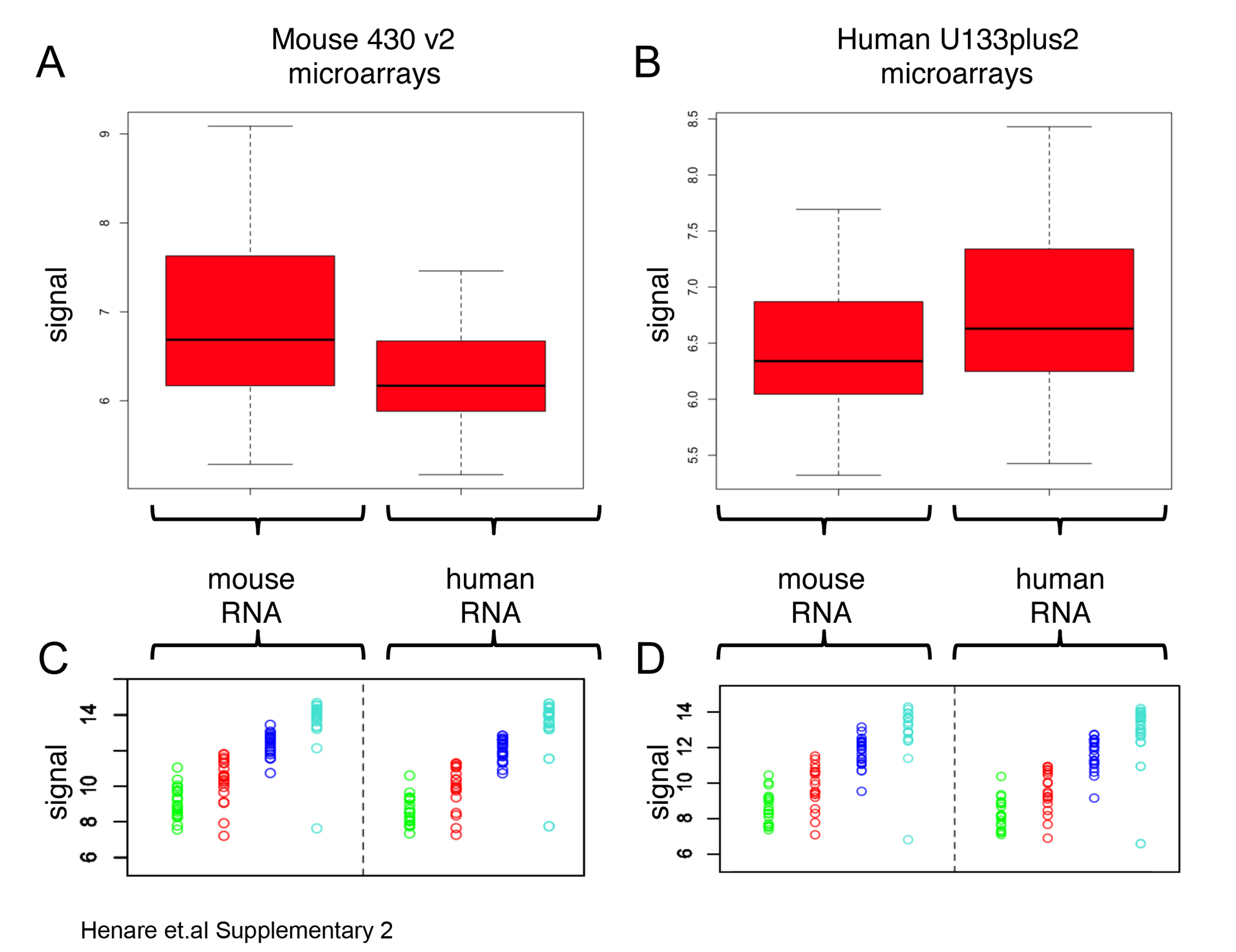

Supplement: Supplementary Figure 2 [file bjc201263x2.tif]

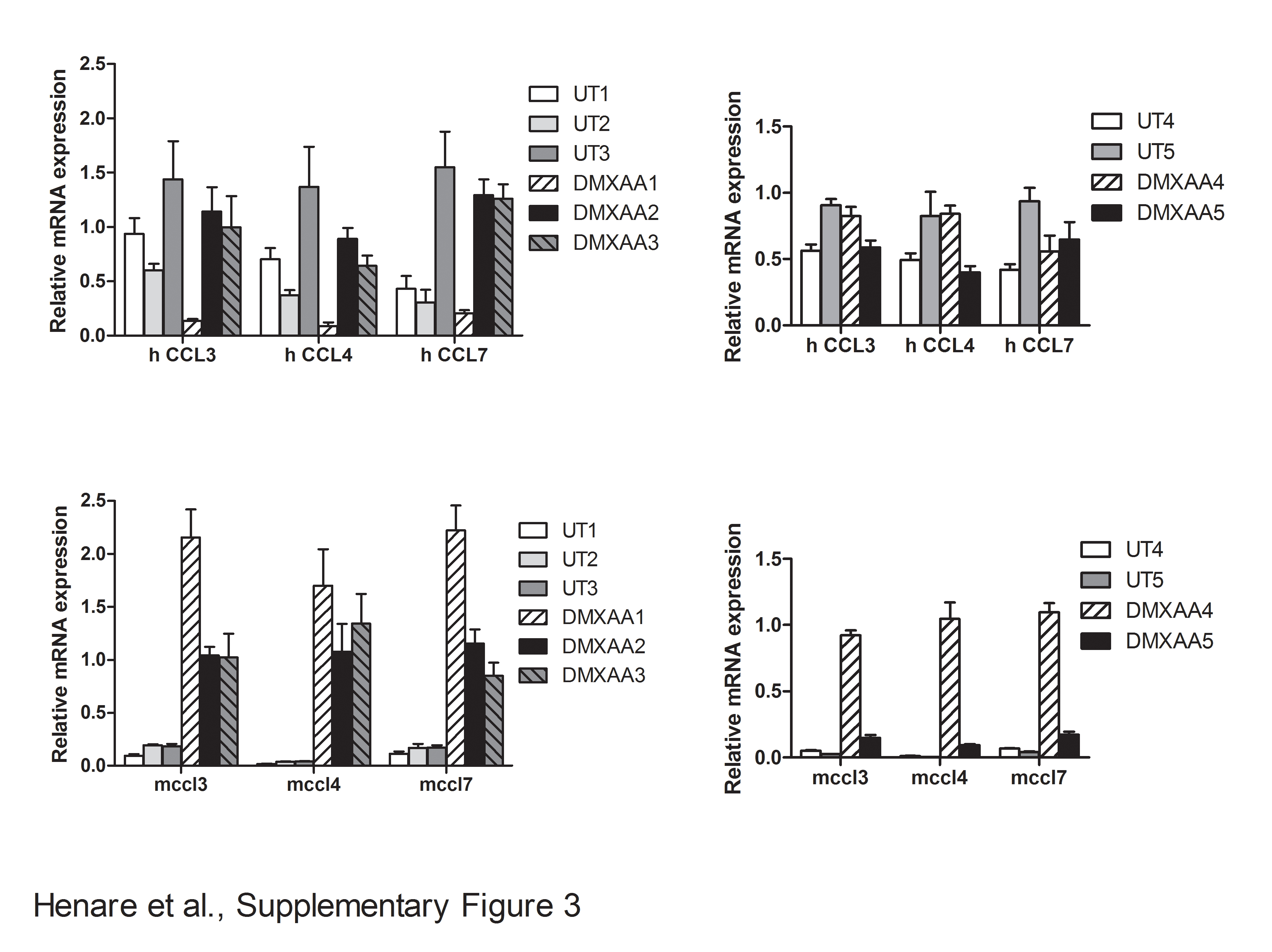

Supplement: Supplementary Figure 3 [file bjc201263x3.tif]
