# Supplementary material for: Dissection of stromal and cancer cell-derived signals in melanoma xenografts before and after treatment with DMXAA
Source: Br J Cancer. 2012 Mar 13;106(6):1134–47. doi: 10.1038/bjc.2012.63 (PMC3304430; doi:10.1038/bjc.2012.63)
Supplement: Supplementary Infomation [file bjc201263x9.doc]

**Supplementary Materials and Methods**

***Characterisation of tumour-infiltrating leukocytes***

Two separate panels of antibodies were used to stain i. myeloid and ii lymphoid leukocytes for flow cytometry as described in the tables below.

1. **Myeloid panel of antibodies**

| **Antibody or stain** | **Antigen / purpose** | **Source** |
| --- | --- | --- |
| M1/70.15.11.5-FITC | CD11b - to identify myeloid cells | MiltenyiBiotec, GmbH, BergishGladbach, Germany |
| 1A8-PE | Ly6G - to identify neutrophils | MiltenyiBiotec, GmbH, BergishGladbach, Germany |
| N418-Allophycocyanin (APC) | CD11c - to identify dendritic cells | MiltenyiBiotec, GmbH, BergishGladbach, Germany |
| CI:A3-1- APC/Cy7 | F4/80 - to distinguish between monocytes and mature macrophages | BioLegend,  San Diego, CA, USA |
| 30-F11- PerCP/Cy5.5 | CD45 - to identify all leukocytes | BioLegend,  San Diego, CA, USA |
| DAPI | Nuclear stain to exclude dead cells | Molecular Probes,  Eugene, OR, USA |

1. **Lymphoid panel of antibodies**

| **Antibody or stain** | **Antigen / purpose** | **Source** |
| --- | --- | --- |
| 29A1.4-Alexa Fluor 647 | NKp46 - to identify NK cells | eBiosciences, San Diego, CA, USA |
| 145-2C11-PE | CD3 - to identify T-lymphocytes | BD Pharmingen,  San Diego, CA, USA |
| RM4-5-PE/Texas Red | CD4 - to identify T-lymphocyte subsets | Molecular Probes,  Camarillo, CA, USA |
| 53-6.7-APC/Cy7 | CD8a - to identify T-lymphocyte subsets | BioLegend,  San Diego, CA, USA |
| 6D5-FITC | CD19 - to identify B-lymphocytes | BioLegend,  San Diego, CA, USA |
| RA3-6B2-PE/Cy7 | CD-45R - to identify B-lymphocytes | BioLegend,  San Diego, CA, USA |
| 30-F11- PerCP/Cy5.5 | CD45 - to identify all leukocytes | BioLegend,  San Diego, CA, USA |
| DAPI | Nuclear stain to exclude dead cells | Molecular Probes, Eugene, OR, USA |

***Analysis of control microarray experiments to estimate the potential for cross-species hybridisation of human and mouse probe sets:***

Genechips were normalized to one another at the probe level using a simple linear transformation based on a fitted line through the average of the replicate spike-in control probe sets present on each array (*BioB, BioC, BioD*and*Cre*), which had very similar expression profiles regardless of cross-species or same-species hybridisation (Supplementary Figure 2), similar to our previously described approach . After normalisation, probes were summarized into probe sets using RMA summarization, and all possible human-mouse orthologous probe set combinations were identified from the manufacturer’s annotation files (NetAffix update 30). Probes sets that had the potential to hybridise across species were defined as a probe set signal from a human array hybridised to mouse RNA, or from a mouse array hybridised to human RNA, of magnitude ≥ 0.6 x the mean of the *BioB* probe set signals for the array. The *BioB* probe set is often regarded as the threshold for detection, and the threshold of 0.6 x *BioB* was an arbitrary choice made to conservatively identify probe sets that show any trend towards cross–species hybridisation. When identifying transcripts that were unlikely to cross-species hybridise, RNAs were defined to be present in a species and therefore to be capable of cross-species hybridising if their same-species hybridisation signal ≥ the 3’ *BioB* signal.

***Permutation analysis to assess the likelihood of identifying gene lists that appear to be enriched for molecular pathways due to chance alone:***

10,000 random lists of genes, which were each the same length as our list of DMXAA-regulated genes, were randomly chosen from the Affymetrix arrays. The intersection between any gene set defined by IPA (e.g. the set of NFκB target genes identified by IPA) and each of these 10,000 randomly chosen gene lists was calculated, and plotted as a histogram using R. The proportion of our 10,000 randomly chosen gene lists that contained more genes from the gene set defined by IPA than our DMXAA-regulated gene list can be viewed as an empirical p-value that approximates the type I error rate.

***Comparison of gene expression in A375 cells in tissue culture and in xenografts***

A375 cells are used in both tissue culture and in xenografts to provide cellular models of malignant melanoma. Therefore, to identify whether specific molecular pathways are differentially activated in these two models we identified RNAs differentially expressed between A375 cells in tissue culture (n=5) and in the untreated xenografts (n=5). We found 657 differentially expressed probe sets (p≤0.05 and ≥1.5-fold up- or down-regulated; Supplementary File 4). There was relatively higher expression in tissue culture of: (i) enzymes related to oxidative stress defences (glucose-6-phosphate dehydrogenase and superoxide dismutase 1), (ii) numerous metabolic enzymes and ubiquitin-conjugating enzymes and several transmembrane receptors including integrin subunits. There was relatively higher expression in the xenografts of extracellular matrix components including: versican, collagens and fibronectin. The implications of these differentially expressed sets of genes are detailed in the Discussion.

***Potential for tumour-stromal cross-talk***

Given the caveat that mouse receptors may not bind human ligands and *vice versa*, we wished to identify potential cross-talk between soluble proteins secreted by stromal cells and their receptors on tumour cells, and between soluble proteins secreted by tumour cells and receptors on stromal cells. To do this we constructed a database of 352 known interactions between secreted proteins and their receptors using the published literature and the IPA database (www.ingenuity.com). We then identified which of these interactions were in theory possible in the A375 xenografts based on the expression above background levels (above mean *BioB* signal) of RNAs encoding these secreted proteins and their receptors (data from 5 untreated and 5 treated tumours. We hoped that this analysis would produce hypotheses about the action of DMXAA for further investigation. For example, based on gene expression above background levels, we found a potential for signalling between 85 soluble factors derived from stromal cells and their ligands on tumour cells in untreated xenografts. An identical analysis of DMXAA-treated xenografts identified a set of 86 potential interactions, 75 of which intersected with the potential interactions identified in the untreated tumours. Some potential interactions were identified only after DMXAA treatment (e.g. proteins encoded by stromal cell-derived factors *CCL3*(MIP-1α) and *CCL7*(MCP-3) with the tumour cell receptor *CCL5*, proteins encoded by stromal *IFNB1* with tumour cell receptors encoded by *IFNAR1*, *IFNAR2* and *TYK2*, as well as stromal cell-derived IL6 with tumour cell receptor IL6ST (=GP130).

***Supplementary Discussion***

Somestudies thatpreviously analysedxenografts using Affymetrix microarrays have not closely assessed whether Affymetrix probe sets cross-species hybridise . This may be justified in-part by the view that Affymetrix microarrays are unlikely to be highly susceptible to cross-specieshybridisation due to their design, including the over-representation of probes from 3’ UTR sequences, in which there is greater than 10% DNA divergence between mouse and human sequences. Studies that have investigated Affymetrix human-mouse cross-species hybridisation have found that it is relatively uncommon. Based on results of a control experiment designed to identify cross-species hybridising probe sets, we flagged 16 % and 14 % of mouse 430v2 and human U133plus2 probe sets, respectively, as clearly capable of human-mouse cross-species hybridisation. These percentages are significantly more conservative than the 4.4% of Affymetrix probe sets identified as cross-species hybridisers by Samuels and co-workers , and the 6% identified by both Naef and Huelsken and by Wilson and colleagues . However, our cross-species hybridisation control experiment was based on small numbers of microarrays and may be specific for the RNA profiles of the cell types we were studying; therefore it will require careful use outside of this context.

**References**

Becker M, Sommer A, KrÃ¤tzschmar JrR, Seidel H, Pohlenz H-D, Fichtner I (2005) Distinct gene expression patterns in a tamoxifen-sensitive human mammary carcinoma xenograft and its tamoxifen-resistant subline MaCa 3366/TAM. *Molecular Cancer Therapeutics***4**(1)**:** 151-170

Creighton C, Kuick R, Misek D, Rickman D, Brichory F, Rouillard J-M, Omenn G, Hanash S (2003) Profiling of pathway-specific changes in gene expression following growth of human cancer cell lines transplanted into mice. *Genome Biology***4**(7)**:** R46

Daniel VC, Marchionni L, Hierman JS, Rhodes JT, Devereux WL, Rudin CM, Yung R, Parmigiani G, Dorsch M, Peacock CD, Watkins DN (2009) A Primary Xenograft Model of Small-Cell Lung Cancer Reveals Irreversible Changes in Gene Expression Imposed by Culture In vitro. *Cancer Research***69**(8)**:** 3364-3373

Hull ML, Escareno CR, Godsland JM, Doig JR, Johnson CM, Phillips SC, Smith SK, Tavare S, Print CG, Charnock-Jones DS (2008) Endometrial-Peritoneal Interactions during Endometriotic Lesion Establishment. *Am J Pathol***173**(3)**:** 700-715

Morimoto AM, Tan N, West K, McArthur G, Toner GC, Manning WC, Smolich BD, Cherrington JM (2004) Gene expression profiling of human colon xenograft tumors following treatment with SU11248, a multitargeted tyrosine kinase inhibitor. *Oncogene***23**(8)**:** 1618-1626

Naef F, Huelsken J (2005) Cell-type-specific transcriptomics in chimeric models using transcriptome-based masks. *Nucleic Acids Research***33**(13)**:** e111

Samuels A, Peeva V, Papa R, Firth M, Francis R, Beesley A, Lock R, Kees U (2010) Validation of a mouse xenograft model system for gene expression analysis of human acute lymphoblastic leukaemia. *BMC Genomics***11**(1)**:** 256

Wilson CL, Sims AH, Howell A, Miller CJ, Clarke RB (2006) Effects of oestrogen on gene expression in epithelium and stroma of normal human breast tissue. *Endocr Relat Cancer***13**(2)**:** 617-628

**Captions for Supplementary Figures**

**Supplementary Figure 1.**(A) Representative FSC/SSC profiles displaying the distribution of cells in the light scatter according to size (FSC) and intracellular granulation (SSC), and showing doublet exclusion: P1 gate determined based on the distribution of CD45^+^ cells after back-gating (not shown). Doublets were excluded after P2- and P3-gating from pulse height and width for FSC and SSC respectively. (B-E) Subsequent analyses performed on P3-gated singlet events. Rows B-E: Dot-plots and corresponding histograms of unstained controls (red) and stained samples (blue). Plots are arranged in columns based on treatment: untreated, and 1, 3, and 7 days after treatment with DMXAA (25 mg/kg) from left to right. Plots B-D represent samples stained with the myeloid panel of antibodies, E represents samples stained with the lymphocyte panel of antibodies.

(B) Singlet (P3-gated) events analysed according to live/dead discriminator (DAPI) staining and expression of CD45 (leukocyte common antigen). Numbers in top left quadrant indicate total counts for DAPI^-^ (Live) CD45^+^ cells.

(C-D) Live CD45^+^ tumour infiltrating leukocytes (TILs) were analysed according to surface expression of CD11b and CD11c (C), and CD11b^+^/CD11c^-^ events were subsequently analysed according to surface expression of granulocytic marker Ly6G and mature macrophage marker F4/80(D). Numbers indicate the percentage of parent events for the corresponding quadrant.

(E) Live CD45^+^ TILs analysed according to surface expression of CD3 and CD19 (not shown), and then CD3^-^/CD19^-^ events analysed according to NKp46 and CD45R. Numbers indicate the percentage of parent events for the corresponding quadrant.

**Supplementary Figure 2.**Labelled human and mouse RNAs were hybridised to both human U133plus2 (A and C) and mouse 430 v2 (B and D) Affymetrixgenechips. Boxplots (A and B) show the signal distributions of all probes on the chips before normalization or summarization; the same-species and cross-species hybridisations produced different data distributions. Graphs (C and D) show the distribution of the replicate hybridisation spike probes, for these spike probes the same-species and cross-species hybridisations produced similar data distributions. Therefore, these spike probes were treated as invariant genes and chips were normalised to one another at the probe level using a simple linear transformation based on a fitted line through the average of these replicate spike probes. These were coloured as follows: *BioB*=green*, BioC*=red*, BioD*=dark blueand*Cre*=sky blue. The normalized data from these chips were then used to estimate to potential of each probe set on the human and mouse chips for cross-species hybridisation.

Supplementary Figure 3.qRT-PCR of xenograft samples by using species-specific primers. The expression level (y-axis) of mouse (A and B) and human (C and D) *CCL3, CCL4*and *CCL7* RNAs were measured relative to invariant controls using species-specific quantitative RT-PCR. UT1, UT2, UT3, UT4 and UT5 represent the five untreatedxenograft samples, while DMXAA1, DMXAA2, DMXAA3, DMXAA4 and DMXAA5 represent the five DMXAA-treatment xenograft samples. Panel A and C represents different batch experiment from panel B and D.

**Supplementary File 1.** The probe sets we estimated were likely to cross-species hybridise on mouse 430 v2 and human U133plus2 Affymetrixgenechips are shown in columns A and B, respectively. The probe sets we estimated were unlikely to cross-species hybridise on mouse 430 v2 and human U133plus2 Affymetrixgenechips are shown in columns C and D, respectively.

**Supplementary File 2.**The 264 mouse probe sets differentially expressed between control and DMXAA-treated cells so that p ≤0.05 and fold change of ≥1.5 up or down are listed. The probe ID and official green symbol (OGS) are shown in columns A and B, respectively. The type of protein encoded by the RNA (identified using the IPA database) is shown in column C, the file is sorted based on this column. Fold change and p-value are shown in columns D and E, respectively. Probe sets that we identified from the control experiments as likely to cross hybridise or unlikely to cross-species hybridise are indicated by dots in columns F and G, respectively.

**Supplementary File 3.**The 262 human probe sets differentially expressed between control and DMXAA-treated cells so that p ≤0.05 and fold change of ≥1.5 up or down are listed. The probe ID and official green symbol (OGS) are shown in columns A and B, respectively. The type of protein encoded by the RNA (identified using the IPA database) is shown in column C, the file is sorted based on this column. Fold change and p-value are shown in columns D and E, respectively. Probe sets that we identified from the control experiments as likely to cross hybridise or unlikely to cross-species hybridise are indicated by dots in columns F and G, respectively.

**Supplementary File 4 –** Comparison of gene expression in A375 cells in tissue culture and in xenografts. 657 Affymetrix probe sets were identified that were differentially expressed (p≤0.05 and ≥1.5-fold up- or down-regulated) between A375 cells in tissue culture and in xenograft tumours. Columns A-G of this file shows respectively: the Affymetrix probe set ID, official gene symbol, gene name, cellular location of encoded protein, type of encoded protein, fold change (on log2 scale) and q value (p value adjusted to control false discovery using Benjamini Hochberg method).

**Supplementary File 5 –**Bioinformatic assessment of the potential for tumour-stromal cross-talk based on gene expression. Combinations of RNAs expressed above background levels in the tumour and stromal cells of treated xenografts (average of n=5) and untreated xenografts (average of n=5) were compared to 352 previously-identified interactions between secreted proteins and their membrane receptors. Column A shows the RNAs encoding proteins secreted into the extracellular space that have previously been shown to bind to or activate the plasma membrane receptors listed in column B. Columns C and D show the types of secreted proteins and membrane receptors that are encoded by the RNAs listed in columns A and B, respectively. Columns E-L show 1 if the RNA is expressed above background levels in the condition indicated, and 0 if expressed below background levels. As indicated by the column headers, columns E-H show the expression in un-treated xenografts, while columns I-L show the expression in DMXAA-treated xenografts. Columns E, F, I and J indicate the expression of RNAs encoding proteins secreted into the extracellular space while columns G, H, K, and L indicate the expression of RNAs encoding membrane receptors. Columns E, G, I and K indicate the expression of RNAs by stromal cells, while columns F, H, J and L indicate the expression of RNAs by tumour cells. Sorting this matrix using a spreadsheet application can identify the potential, based on RNA expression information, for stroma-tumour, tumour-stroma, stroma-stroma and tumour-tumour cross talk.
